# Different Brain Regions are Infected with Fungi in Alzheimer’s Disease

**DOI:** 10.1038/srep15015

**Published:** 2015-10-15

**Authors:** Diana Pisa, Ruth Alonso, Alberto Rábano, Izaskun Rodal, Luis Carrasco

**Affiliations:** 1 Centro de Biología Molecular “Severo Ochoa”. c/Nicolás Cabrera, 1. Universidad Autónoma de Madrid. Cantoblanco. 28049 Madrid. Spain; 2 Department of Neuropathology and Tissue Bank, Unidad de Investigación Proyecto Alzheimer, Fundación CIEN, Instituto de Salud Carlos III, Madrid. Spain

## Abstract

The possibility that Alzheimer’s disease (AD) has a microbial aetiology has been proposed by several researchers. Here, we provide evidence that tissue from the central nervous system (CNS) of AD patients contain fungal cells and hyphae. Fungal material can be detected both intra- and extracellularly using specific antibodies against several fungi. Different brain regions including external frontal cortex, cerebellar hemisphere, entorhinal cortex/hippocampus and choroid plexus contain fungal material, which is absent in brain tissue from control individuals. Analysis of brain sections from ten additional AD patients reveals that all are infected with fungi. Fungal infection is also observed in blood vessels, which may explain the vascular pathology frequently detected in AD patients. Sequencing of fungal DNA extracted from frozen CNS samples identifies several fungal species. Collectively, our findings provide compelling evidence for the existence of fungal infection in the CNS from AD patients, but not in control individuals.

Neurodegenerative diseases constitute a heterogeneous group of disorders of the central nervous system (CNS) that are characterised by a slow and irreversible loss of neuronal functions. The aetiology of primary neurodegenerative diseases, such as Alzheimer’s disease (AD), multiple sclerosis (MS), Parkinson’s disease (PD) and amyotrophic lateral sclerosis (ALS), remains largely unknown. A common feature of many neurodegenerative diseases is the presence of aggregates of misfolded proteins (intracellular inclusions) in regions of the CNS that can serve as neuropathological hallmarks for disease diagnosis^1^,^2^. Depending on the particular disease, these insoluble fibrillar aggregates can vary in distribution and composition^3^. Histopathologically, AD is characterised by the accumulation of intracellular tangles of hyperphosphorylated tau protein and extracellular deposits of amyloid protein^4^,^5^. Proteolytic processing of membrane-associated amyloid precursor protein (APP) results in the generation of neurotoxic amyloid β (Aβ) peptide^6^,^7^, which is the major component of the distinctive senile plaques in AD. The cytotoxicity induced by Aβ pepetide involves disruption of calcium homeostasis, oxidative stress, synaptic dysfunction and neuronal loss^8^,^9^,^10^. The prevailing dogma to explain the pathogenesis of AD is that the accumulation of amyloid deposits formed by Aβ pepetide may induce intracellular tangles of tau protein that in turn leads to neuronal death^11^. However, the so-called “amyloid hypothesis” has been questioned by several findings including the failure of clinical trials aimed to lower amyloid deposits or tau tangles^12^,^13^,^14^. Moreover, many elderly people with normal cognitive function have substantial amyloid burden in their CNS^11^. At present, there is no therapy to stop or reverse the symptoms of AD. Aside from cognitive decline, the vast majority of AD patients present clear signs of inflammation and damage to blood vessels^15^,^16^. Inflammation of the CNS and immune activation play a major role in the pathophysiology of AD. Indeed, a number of cytokines, such as interleukins (IL-1 and IL-6), tumor necrosis factor α and interferon γ, are elevated in the brain of AD patients, suggesting an increased immune response^17^,^18^,^19^. These observations have led to the speculation that AD has an autoimmune aetiology^20^. Many investigators have also considered the idea that AD is an infectious disease, or at least that infectious agents constitute a risk factor for AD^21^,^22^,^23^. Accordingly, genetic material from several viruses and bacteria have been reported in brains from AD patients. In particular, herpes simplex type 1 (HSV-1) and *Chlamydophila pneumoniae* have been suggested as potential aetiological agents of AD. In addition, brain infection by several pathogens may induce amyloid formation^24^,^25^,^26^. Furthermore, Αβ peptide exhibits antimicrobial activity and shows particularly strong inhibitory activity against *Candida albicans*^27^.

Recently, we provided strong evidence for fungal infection in AD patients^28^,^29^. Fungal DNA and proteins were found in frozen brain tissue from AD patients, but not from control patient tissue. Moreover, fungal material could be detected intra- and extracellularly in neurons from AD patients. In the present work, we have examined in detail the presence of fungal structures in different regions of the brain of an AD patient by immunohistochemistry. No fungal material was observed in brain tissue from ten control individuals, whereas fungal infection was clearly present in brains from ten additional AD patients. Moreover we were able to amplify fungal DNA from frozen tissue of different AD brain regions. Collectively, our findings provide compelling evidence for the presence of fungal infection in brains from all AD patients analysed.

## Results

### Fungal structures in AD CNS

**Figure 1:**
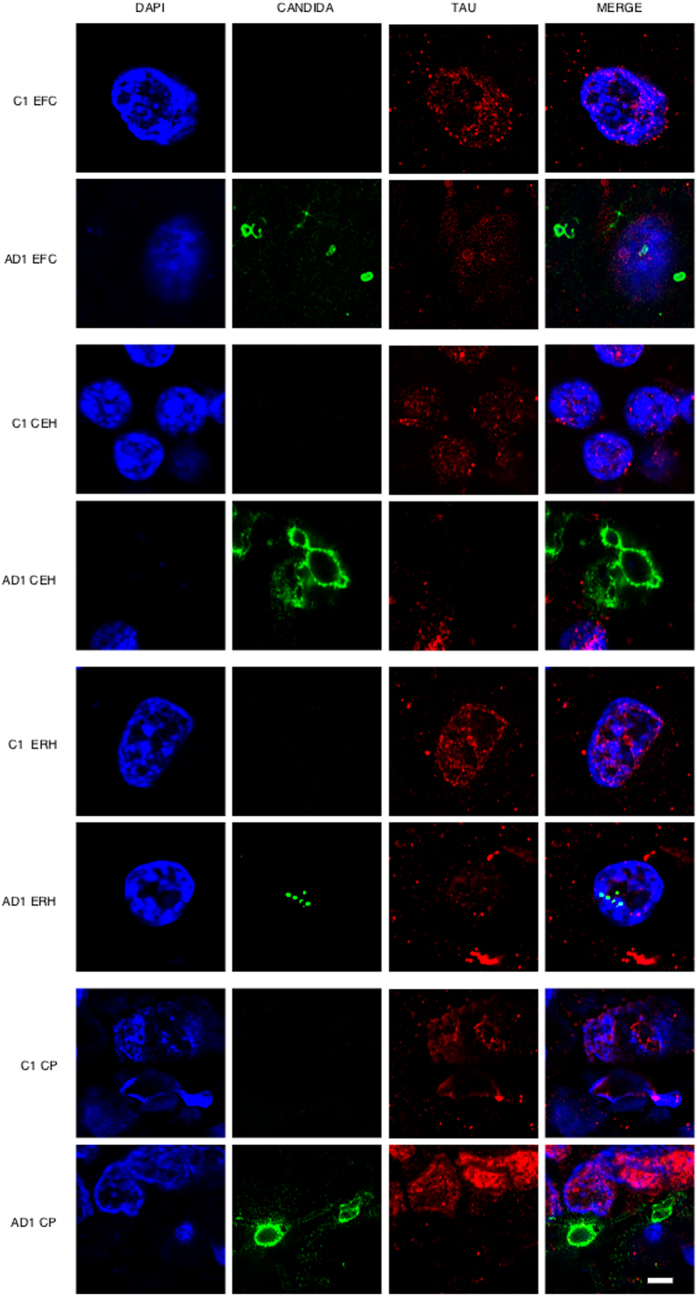
Immunohistochemistry analysis of tissue sections from different regions of the CNS using anti-*C. glabrata* antibodies. CNS sections from patient AD1 and control individual C1 were obtained from fixed tissue and immunohistochemistry analysis by confocal microscopy was carried out as detailed in Materials and Methods. EFC: external frontal cortex; CEH: cerebellar hippocampus; ERH: entorhinal cortex/hippocampus; CP: choroid plexus. DAPI appears in blue, anti-*C. glabrata* is shown in green and Tau_T100_ in red. The different panels in the figure are indicated. Scale bar: 5 μm.

One of the most direct approaches to detect fungal infection in the CNS is the visualisation of fungi in tissue sections. It is well established that the hierarchical pattern of neurofibrillary degeneration and thus the early pathological lessions in AD patients generally starts by modifications in the entorhinal cortex, followed by the hippocampus, association cortex and finally the primary neocortex^11^. We first analysed fixed sections from different regions of the CNS from one AD patient (AD1) and a control individual (C1) (Supplementary Table I). The regions examined were external frontal cortex (EFC), cerebellar hemisphere (CEH), entorhinal cortex/hippocampus (ERH) and choroid plexus (CP). Notably, fungal cells were detected in the four regions examined from patient AD1 as demonstrated by immunohistochemistry and confocal microscopy using anti-*C. glabrata* antibodies (Fig. 1). In some instances, fungal cells were clearly visible inside neurons and exhibited an intranuclear location as indicated by counterstaining with the DNA stain, 4'6-diamidino-2-phenylindole (DAPI). The size of the fungal bodies was variable; in some instances, the size was 1–2 μm, whereas the diameter of other fungal bodies was greater (approximately 5–10 μm). In other instances, smaller fungal bodies of 0.4–1 μm were evident depending on the field analysed. The 0.4–1 and the 1–2 μm-sized bodies are similar to those previously reported for some intracellular yeast cells^30^,^31^,^32^. These intracellular forms are known as endomycosomes^29^,^33^. Endothelial cells in the CP may also contain fungal bodies. No fungal cells or fungal material were apparent in the different CNS regions from the control (C1) individual (Fig. 1). Curiously, immunostaining of tau protein with specific antibodies localised tau not only in the cytoplasm, but also in the nucleus in both AD1 and C1 sections. This finding is consistent with the observation that nuclear pores are damaged in elderly people, particularly with neurodegenerative diseases, and cytoplasmic proteins can relocate to the nucleus^34^,^35^. Nuclear tau protein staining was very strong in neurons where intranuclear fungal bodies were detected.

Wider fields illustrating the presence of additional fungal bodies and a more general view of the fungal infection are shown in Supplementary Figures 1, 2 and 3. Nuclear (DAPI) staining (blue) and double immunofluorescence staining to detect fungal structures (green) and tau protein (red) was carried out and only the merged panel is shown for space restrictions. Several fungal morphologies could be observed in the EFC, with sizes ranging from 0.4–1 and 5–10 μm. The sizes of the fungal bodies found in the CEH were approximately 1–2 μm. Strikingly, two different fields of the ERH revealed amylaceous bodies (corpora amylacea), which were prominently stained in the border of these rounded structures. However, immunostaining was not evenly distributed in all the border zones, indicating that the fungal material is not distributed homogeneously. Collectively, these observations demonstrate the presence of fungal material in different CNS regions examined, but only in the AD patient (AD1). The fungal structures could be detected outside and inside nuclei and, in some instances, fungal cells were positive for DAPI, indicating that they contain nucleic acids (see upper-right panel in Supplementary Figure 1).

### Detection of fungal cells and hyphae using different anti-fungal antibodies

**Figure 2:**
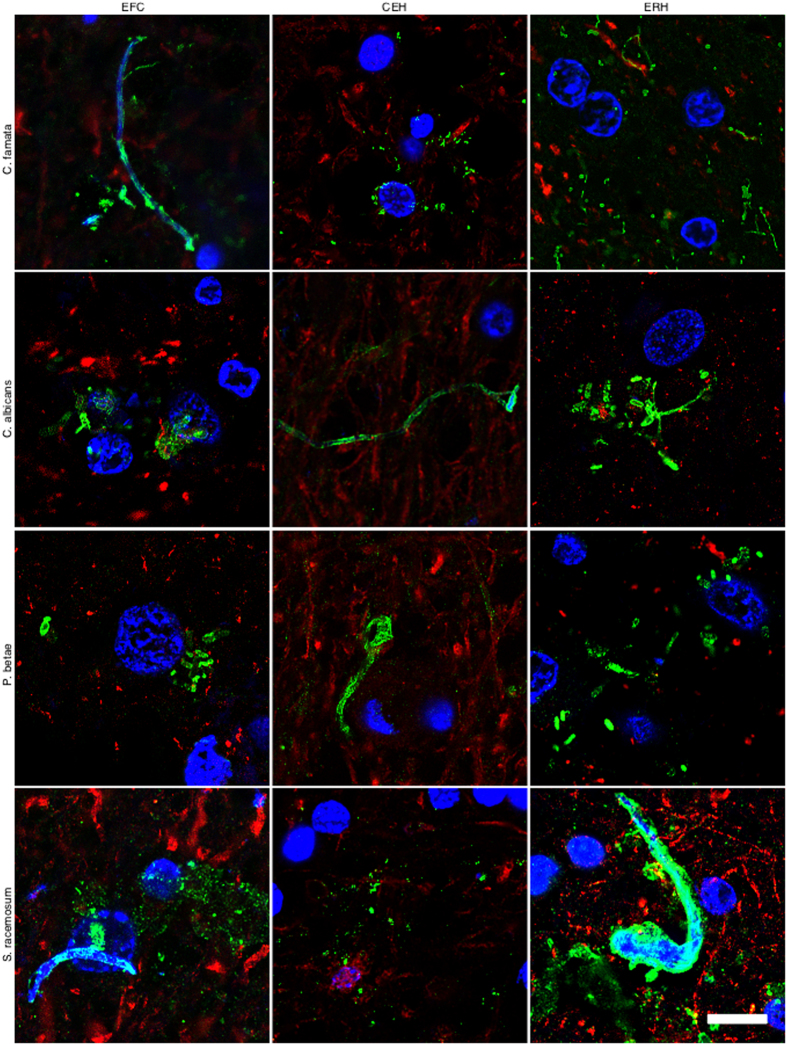
Immunohistochemistry analysis of CNS sections from patient AD1 using different anti-fungal antibodies. Immunohistochemistry analysis of different CNS sections from patient AD1 was carried out as indicated in Fig. 1. EFC: external frontal cortex; CEH: cerebellar hippocampus; ERH: entorhinal cortex/hippocampus. DAPI appears in blue, anti-*C. famata*, anti-*C. albicans*, anti-*P. betae* and anti-*S. racemosum* are shown in green and human neurofilament in red. The different panels in the figure are indicated. Scale bar: 10 μm.

It must be kept in mind that the immunoreactivity observed with anti-*C. glabrata* antibodies does not necessarily mean that this yeast is present. Because the antibodies employed are rabbit polyclonal, they can crossreact with a number of proteins from other fungi. The spectrum of proteins recognised by the different anti-fungal antibodies employed in this work vary and depend on the fungal species present in each case. However, these anti-fungal antibodies do not crossreact with cellular proteins from control individuals. To further assess whether fungal cells were present in patient AD1, we carried out immunohistochemistry analysis using rabbit polyclonal antibodies raised against other fungi. Four additional antibodies (raised against *C. famata, C. albicans. P. betae*, and *S. racemosum*) detected fungal components (green) in the tissue sections analysed (Fig. 2), as demonstrated by double staining with an anti-neurofilament antibody (red). As indicated previously, the size of the fungal cells detected with the antibodies varied. The additional antibodies also detected long fibrilar structures clearly resembling fungal hyphae, with sizes ranging from 0.1 μm to 1–2 μm. The variety of sizes and morphologies observed in these sections using different anti-fungal antibodies is consistent with the notion that several fungal species were present in the three CNS regions examined. Analyses of tissue sections from C1 using these antibodies failed to reveal any fungal material (results not shown). The possibility that these antibodies recognise human proteins present only in CNS samples from patient AD1 and form structures that resemble different fungal morphologies is unlikely. To further test the presence of fungal proteins in patient AD1, we extracted proteins from different CNS regions of AD1 and C1 and performed western blotting with anti-*C. albicans* antibodies (Supplementary Figure 4). No specific protein bands were detected by this technique, most probably due to the fact that the amount of fungal proteins is extremely low. To unequivocally identify fungal proteins in brain tissue from AD patients, proteomic methodologies are required as we have previously reported^28^. Using this approach, we have detected several fungal proteins that are present in CNS samples from AD patients, but not in control individuals.

**Figure 3:**
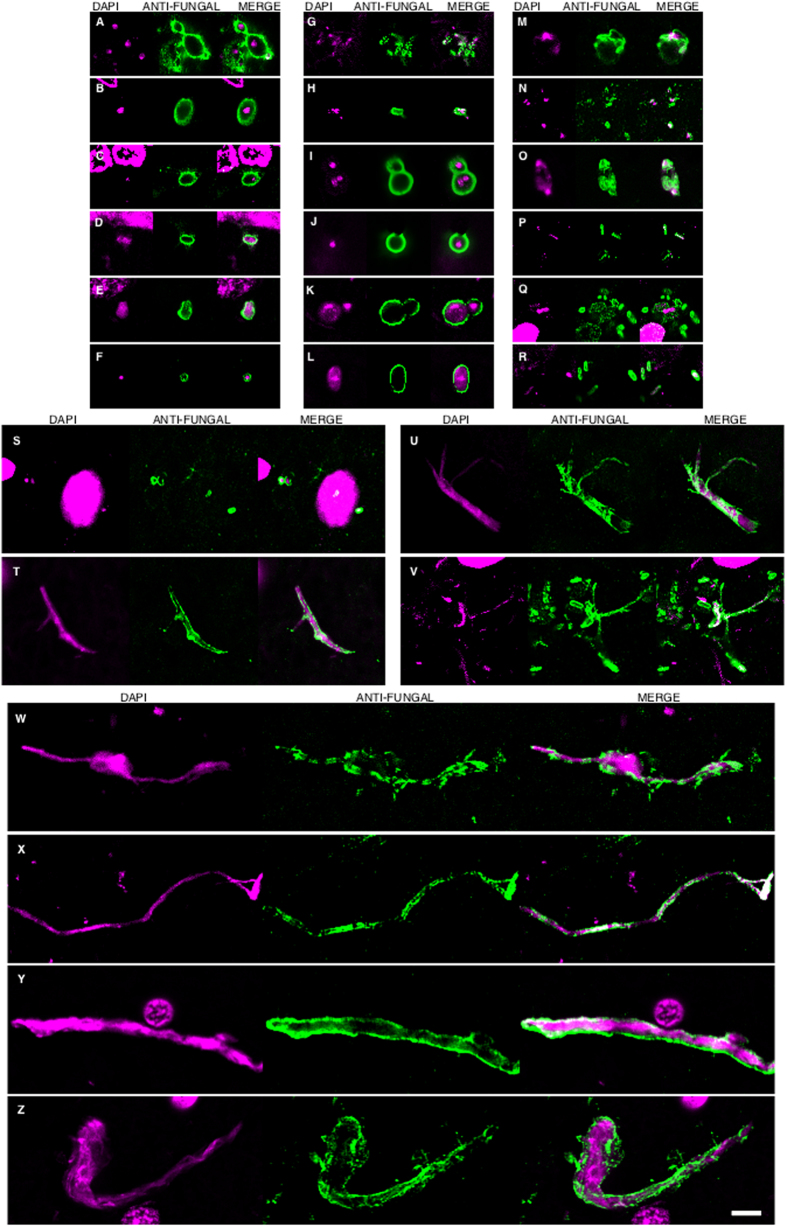
DAPI staining of nuclei of the different fungal morphologies. DAPI staining is shown in magenta to visualize more clearly the fungal nuclei. Immunoreactivity with the fungal antibodies indicated is shown in green. Panels A–G and S: anti-*C. glabrata* as primary antibody. Panels H–J, T, U, W: anti-*C. famata* as primary antibody. Panels: K–M, V, X: anti-*C. albicans* as primary antibody. Panels N, O, Y, Z: anti-*S. racemosum*. as primary antibody. Panels P-R: anti- *P. betae* as primary antibody. Panels K, L, S, T, U, Y: EFC of patient AD1. Panels A, H, M–Q, X: CEH of patient AD1. Panels I, J, R, V, W, Z: ERH of patient AD1. Panels B, C, D: CP of patient AD1. Panel E: ERH of patient AD3. Panel F: ERH of patient AD4. Panel G: ERH of patient AD9. Scale bar: 5 μm is the same for all panels shown in the figure.

In accord with the earlier finding, some fungal cells and hyphae stained positive for DAPI (blue), suggesting that they contain nucleic acids (Fig. 2). In EFC sections stained with anti-*C. famata* and anti-*S. racemosum* antibodies, DAPI staining was clearly located inside hyphae. Indeed, DAPI positivity could be seen in the vast majority of cells and hyphae after longer exposure times, but under these conditions neuron nuclei were overexposed. To overcome this limitation, the blue DAPI staining was converted to magenta and the antifungal antibody staining was in green. Under these conditions, positive DAPI staining was observed in all the morphologies found, i.e. yeast-shaped cells and hyphae (Fig. 3), and this was also the case when the anti-*C. glabrata* antibodies were employed (Fig. 1).

**Figure 4:**
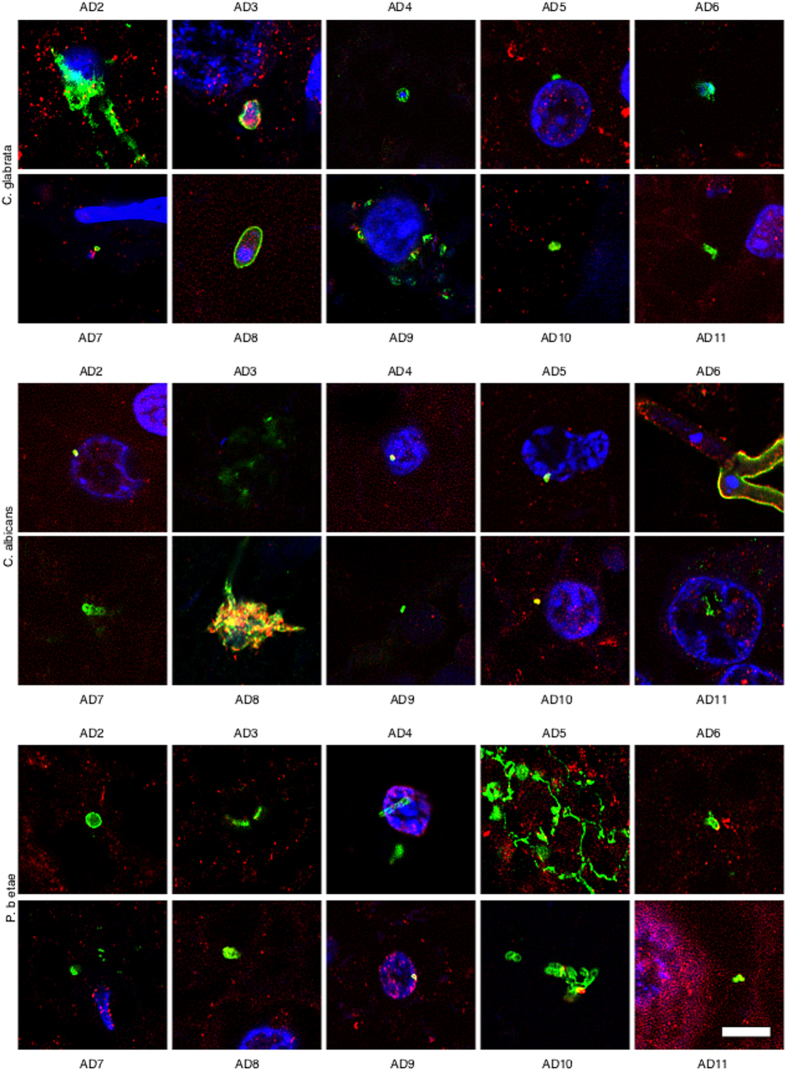
Immunohistochemistry analysis of entorhinal cortex sections from ten different AD patients. Entorhinal cortex sections from ten different AD patients were incubated with different antibodies (anti-*C. glabrata*, anti-*C albicans* and anti-*P. betae*) and are shown in green; human α-tubulin immunostaining is shown in red. Double immunofluorescence assay and confocal microscopy was carried out as indicated in Fig. 1 and Materials and Methods. DAPI appears in blue. Scale bar: 5 μm.

Because most of the above results were obtained from only one AD patient and one control individual, it was of interest to examine CNS sections from additional AD patients and controls. To this end, we analysed ERH tissue sections from a further ten AD patients and ten controls by double immunostaining with anti-fungal (green) and anti-tubulin (red) antibodies. Notably, fungal infection was evident in all AD patients studied (Fig. 4), whereas no fungal cells were detected in tissue sections from control individuals (Supplementary Figure 5). The morphology of the fungal structures detected in the additional AD patients was similar to that described for AD1, although not all structures were found in all patients. Moreover, some of these structures were very striking; for example, conidial structures were observed in patients AD2 (anti-*C. glabrata*) and AD8 (anti-*C. albicans*), and hyphae formation was observed from a fungal cell in patient AD6 (anti-*C. albicans*). The small hyphae and yeast cells found in patient AD5 (anti-*Phoma* staining) was very striking. The existence of different fungal morphologies reinforces the idea that several species can be present, supporting the concept of mixed fungal infections. In conclusion, fungal cells and/or hyphae were found in all AD patients analysed although the morphological characteristics may be different for each patient, thus implying that the fungal species present in each patient may also differ.

### Fungal infection of the neurovascular system

**Figure 5:**
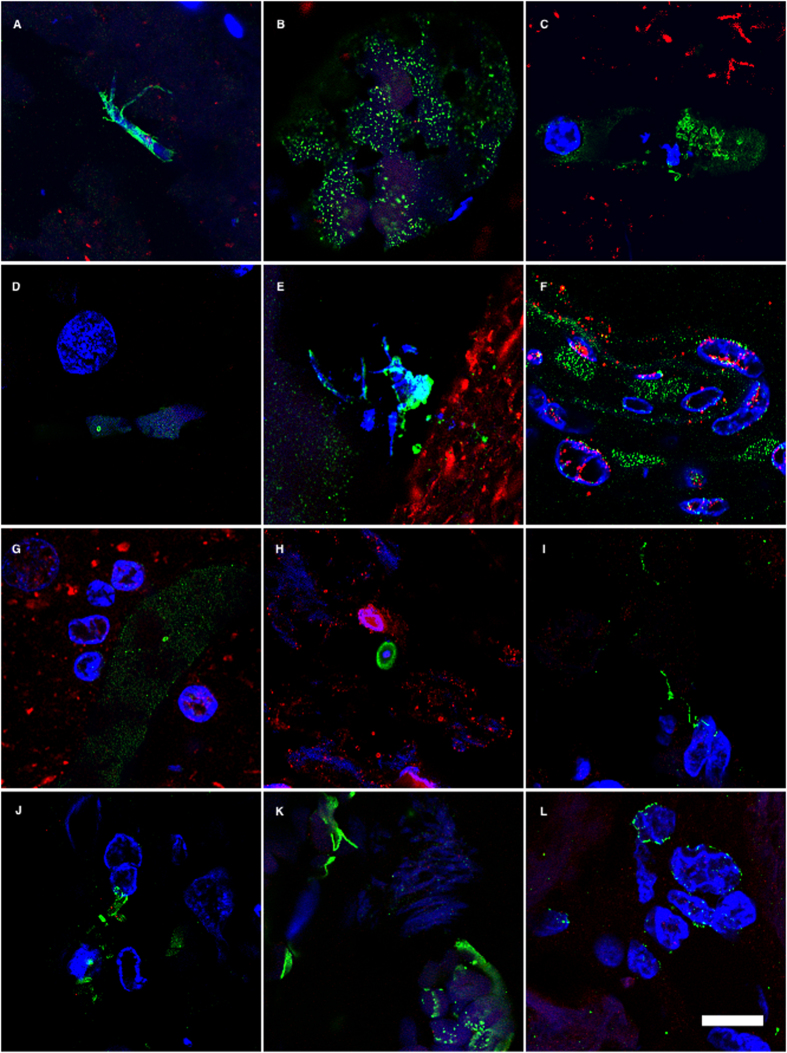
Fungal bodies in CNS blood vessels from patient AD1 detected by immunohistochemistry. Double immunofluorescence assay analyzed by confocal microscopy as described in Materials and Methods. Blood vessels from different CNS regions from patient AD1 are shown. Panels A–E: EFC. Panels F and G: ERH. Panels H–L: CP. Panels A and I: anti-*C. famata* as primary antibody. Panels B, C, J: anti-*C. albicans* as primary antibody. Panels D, G, K: anti-*P. betae* as primary antibody. Panels E and L: anti-*S. racemosum* as primary antibody. Panels F and H: anti-*C. glabrata* as primary antibody. Anti-fungal antibodies are shown in green. An anti-human neurofilament antibody was used in all panels, except panels F and H, which were stained with anti-Tau_T100_ antibodies (red.) DAPI appears in blue. Scale bar: 10 μm.

The majority of AD patients exhibit pathological lesions of the vascular system in the CNS^16^,^36^. Up to 90% of AD patients present various cerebrovascular pathologies, including cerebral amyloid angiopathy, microinfarcts, haemorrhages and microvascular degeneration. Accordingly, deposits of amyloid β in the walls of capillaries, small arterioles and medium-size arteries are evident in most AD patients studied. These amyloid deposits give rise to cerebral amyloid angiopathy. We reasoned that if fungal infection is present in blood vessels, it might induce the formation of amyloid deposits and angiopathy. To assess fungal infection in blood vessels from EFC, ERH and CP regions, we carried out confocal immunofluorescence analysis. Fungal cells of different sizes and hyphae were detected inside capillaries and other blood vessels (Fig. 5). Some staining was also evident in the vessel walls, demonstrating that fungal infection can be detected in the neurovascular system. Also, the CP region from patient AD1 (Fig. 5), but not from C1 (Supplementary Figure 6), contained fungal cells and hyphae that immunoreacted with the different anti-fungal antibodies. These results are in good agreement with the notion that several fungal species can infect blood vessels and cause pathological modifications^37^,^38^,^39^. These studies were extended to the analysis of the CP region from three additional AD patients (AD12, 13, 14) using several anti-fungal antibodies., Consistent with the previous result, fungal material was detected in all three cases examined, but not in the control (Supplementary Figure 6). In conclusion, fungal structures can be observed also in the vascular system of AD patients.

### Identification of fungal species in the different CNS regions

DNA amplification and sequencing is the most precise approach to determine specific fungal species present in CNS tissues. Because the vast majority of DNA from tissue samples is human, we previously developed a sensitive nested PCR assay to amplify fungal DNA present in very minor amounts^28^,^33^,^40^,^41^. We recently used this tecnhnique successfully to identify several fungal species in brain samples from AD patients^40^. To identify the fungal species present in the different brain regions of patient AD1, we extracted DNA from frozen tissue and performed PCR using primers that amplify two internal transcribed spacers (ITS-1 and ITS-2) located between ribosomal RNA genes (see scheme, Supplementary Figure 7). Several different PCR assays were carried out since in our experience the use of several primer pairs ensures the amplification of DNA from different fungal species. We first amplified ITS-1 with primers ITS-1 (external) and then three different PCR assays were subsequently performed with internal primer pairs (ITS-1, internal). In addition, ITS-2 was first amplified with primers ITS-2 (external) followed by a second PCR with an ITS-2 (internal) primer pair. DNA amplified by each PCR was separated on agarose gels and we sequenced the extracted fragments. A typical PCR result after amplification of the ITS-1 using DNA extracted from the different CNS regions of patient AD1 and C1 is shown in Supplementary Figure 7. No PCR products were amplified from DNA extracted from the different regions of C1, or from controls for DNA extraction and PCR. These findings reveal that no fungal contamination occurred in the PCR assay or during DNA extraction. Sequencing of each fragment from the four PCR assays using DNA from patient AD1 revealed a number of fungal species as listed in Table 1. Of note, several species could be detected from the same region, supporting the concept of mixed fungal infection. Conversely, no single fungal species was present in all four regions of the CNS examined. Of note, some of the species detected, such as *Malasezzia spp*., *Phoma* and *S. cerevisiae*, have been previously identified in AD brains^40^. Significantly, the majority of the species listed in Table 1 are described as human pathogens^42^,^43^. The possibility that different fungal species or a combination of species serves as a risk factor or represents the cause of AD might explain the diversity observed in the evolution and severity of clinical symptoms in each AD patient.

## Discussion

A major goal of AD research is to uncover the precise aetiology of the disease in order to implement adequate therapies to halt or even reverse clinical symptoms. The possibility that AD is a fungal disease, or that fungal infection is a risk factor for the disease, opens new perspectives for effective therapy for these patients. The present findings demonstrate that fungi can be detected in brain tissue from different regions of the AD CNS. In all eleven patients (plus three additional CP samples) described in this study, as well as in four patients previously analysed, there is clear evidence for fungal cells inside neurons or extracellularly^29^. Therefore, 100% of the AD patients analysed thus far by our laboratory present fungal cells and fungal material in brain sections. Moreover, fungal macromolecules (polysaccharides, proteins and DNA) have been found in blood serum from AD patients^40^ and fungal proteins and DNA were detected by proteomic analyses and PCR, repectively, from frozen tissue of AD brain^28^. Observations from other laboratories also support the possibility of fungal infections in AD patients. For example, chitin bodies have been found in AD brains^44^,^45^. The proposal that chitin-like polysaccharides play a role in the amyloidogenic process and are aberrantly synthesised by neural cells has been suggested^46^; however, since chitin is a component of the fungal cell wall, it seems possible that these chitin polysaccharides may originate from fungi. Supporting this notion, increased chitinase levels are found in blood serum and cerebrospinal fluid from AD patients^47^,^48^,^49^,^50^. Presumably, the presence of the substrate, i.e. fungal chitin, induces the production of chitinase. Moreover, antifungal treatment in two patients diagnosed with AD reversed clinical symptoms^51^,^52^. The interpretation of these results was that perhaps these patients were misdiagnosed. Interestingly, Aβ peptide has potent antimicrobial activity, particularly against *C. albicans*^27^. Accordingly, it might be possible that the presence of a chronic fungal infection in AD CNS triggers the synthesis of Aβ peptide, which in turn leads to amyloid deposits. These reports together with our present findings support the notion that fungal infection may exist in AD.

Proceeding on the assumption that fungi are the aetiological agent of AD, all of the symptoms observed in AD patients can be readily explained. For example, the slow progression of the disease fits well with the chronic nature of fungal infections if they remain untreated. Moreover, inflammation and activation of the immune system may be due to an infectious fungal agent. Disseminated fungal infections can induce cytokine production^53^,^54^,^55^, which can take place years before the onset of cognitive decline as observed in AD. Thus, this disseminated infection may slowly spread to the CNS and synaptic dysfunction and neuronal loss takes place only when the fungal burden in some areas of the CNS is high. It is quite possible that the existence of one fungal infection may facilitate the colonisation by other fungal species that can affect other areas of the CNS, giving rise to mixed fungal infections. The diversity of fungal species that can affect the CNS, as well as the combinations of these species, may account for the observed differences in the evolution and severity of clinical symptoms found in AD patients. The pathology of the blood vessels observed in most AD patients can also be explained since they can be infected by fungi^37^,^38^,^39^, particularly arteries, where the oxygen content is higher. Finally, the genetic predisposition observed in approximately 2–5% of AD patients can simply be due to their predisposition to acquire fungal infections because the genetic background of each individual is permissive for this^56^,^57^. To the best of our knowledge, none of the symptoms established in AD pathology seem incompatible with the concept that AD may be caused by fungi. Moreover, the dissapointing results obtained in human clinical trials designed to lower the burden of β-peptide or tau tangles does not support the amyloid cascade hypothesis^12^,^13^,^14^. These findings suggest that the main pathological agent in AD is still unidentified.

The existence of fungal infection in AD patients may be due to its involvement in the aetiology of this disease, but it could also be possible that, for reasons yet unknown, these patients are more prone to this type of infection. The fact that they are elderly and may have a poor adaptive immune response, or possibly changes in the diet and hygiene habits, may contribute to the emergence of fungal infections. It is evident that clinical trials will be necessary to establish a causal effect of fungal infection in AD. There are at present a number of highly effective antifungal compounds with little toxicity^58^,^59^,^60^,^61^. A combined effort from the pharmaceutical industry and clinicians is needed to design clinical trials to test the possibility that AD is caused by fungal infection.

## Materials and Methods

### Description of patients and control individuals

In this study, we analysed both frozen and paraffin-fixed samples of tissue obtained from brain donors diagnosed with AD and control individuals. Details about the age and gender of each patient are listed in Supplementary Table I. All samples were supplied by Banco de Tejidos, Fundación CIEN (Centro de Investigación de Enfermedades Neurológicas, Madrid. Spain). The ethics committee of Universidad Autónoma de Madrid approved the study. The transfer of samples was performed according to national regulations concerning research on human biological samples. For all cases, written informed consent is available. All ethico-legal documents of the brain bank, including written informed consent, have been approved by an ethics committee external to the bank. The donors were anonymous to the investigators who participated in the study.

### Antifungal antibodies

Fungi were cultured as described^29^,^33^. After autoclaving and lyophilisation of fungal cells, rabbit antisera against *Candida famata*, *C. albicans*, *C. glabrata*, *Phoma betae and Syncephalastrum racemosum* were obtained by inoculation of 1 or 2 mg of dried fungi in 0.5 ml PBS, previously mixed with an equal volume of Freund’s adjuvant. Rabbits were inoculated up to three times every three weeks and the antibody titre and specificity of the sera were tested by immunohistochemistry and immunoblotting using fungal proteins. The protocols employed were approved by the ethics committee of Centro de Biologia Molecular “Severo Ochoa” (identification number: ES280790000180).

### Immunohistochemistry analysis

Tissue sections from the CNS (5 μm) were fixed in 10% buffered formalin for 24 h and then embedded in paraffin following standard protocols. For immunohistochemical analysis, paraffin was removed and tissues were rehydrated and boiled for 2 min in citrate buffer and then incubated for 10 min with 50 mM ammonium chloride. Subsequently, tissue sections were incubated for 10 min with PBS/Triton X-100 (0.1%) followed by 20 min with PBS/BSA (2%). Sections were incubated overnight at 4 °C with a mouse monoclonal antibody raised against human α-tubulin (Sigma), human phospho-PHF-tau, clone AT100 (Thermo Scientific), or human neurofilament protein, clone 2F11 (Dako), all at 1:50 dilution, or a rabbit polyclonal antibody raised against proteins obtained from *C. glabrata, C. famata, C. albicans, P. betae and S. racemosum* at 1:500 dilution. Thereafter, sections were washed with PBS and further incubated for 1 h at 37 °C with donkey anti-mouse IgG secondary antibody conjugated to Alexa 555 (Invitrogen) for α-tubulin, T_T100_ and neurofilament, and donkey anti-rabbit IgG secondary antibody conjugated to Alexa 488 (Invitrogen) at 1:500 dilution for *C. glabrata, C. famata, C. albicans, P. betae and S. racemosum*. Subsequently, tissue sections were stained with DAPI (Merck) and samples were treated with autofluorescence eliminator reagent (Merck). All images were collected and analysed with a LSM710 confocal laser scanning microscope combined with the upright microscope stand AxioImager. M2 (Zeiss) running Zeiss ZEN 2010 software. The spectral system employed was Quasar + 2 PMTs. Images were deconvoluted using Huygens software (4.2.2 p0 Scientific Volume Imaging) and visualised with Fiji/Image J.

### Analysis of proteins by western blotting

Protein extracts of EFC, CEH and ERH of AD1 and C1 were collected in sample buffer, boiled for 5 min and resolved by SDS-PAGE. After electrophoresis, proteins were transferred to a nitrocellulose membrane. Specific rabbit polyclonal antibodies raised against *C. albicans* were used at 1:1000 dilution. Anti-rabbit immunoglobulin G antibody coupled to peroxidase (Amersham) was used at a 1:5000 dilution. Protein bands were visualised with the ECL detection system (Amersham).

### DNA Extraction from frozen CNS tissue

DNA was extracted from frozen samples of different CNS regions using the QIAmp (Qiagen) Genomic DNA Isolation Kit as follows: 20 μl proteinase K (>600 mAU/ml) and 180 μl of buffer ATL were added to 25 mg of brain tissue, followed by pulse-vortexing for 15 s. Digestion was carried out at 56 °C for 1–3 h with agitation. Subsequently, 200 μl of buffer AL was added to each sample followed by vortexing for 15 s and incubation at 70 °C for 10 min. A 200 μl volume of ethanol was then added to each sample followed by vortexing for 15 s. The mixture was applied to the QIAamp Mini spin column and centrifuged at 8000 rpm for 1 min. Then, 500 μl buffer AW was applied to the column followed by centrifugation at 8000 rpm for 1 min. After a final wash step with 500 μl of buffer AW2 (14000 rpm for 3 min), samples were collected in 40 μl distilled water and DNA was quantified in a NanoDrop® ND-1000 UV-Vis spectrophotometer. Negative controls included three samples of tri-distilled filtered water.

### Design of oligonucleotides

For ITS oligonucleotides, the gene sequences of 5.8S, 18S and 28S rRNA as well as internal transcribed spacer regions (ITS-1 and ITS-2) from several organisms (*C. albicans, C. famata*, *C. parapsilosis*, *C. glabrata*, *Rhodotorula mucilaginosa*, *Pichia guilliermondii*, *Cryptococcus neoformans* and *Homo sapiens*) were accessed *via* the GenBank database and were aligned using Clustal W sequence analysis package. Multiple potential primer-binding sites for primer pairs were chosen by comparing regions of *Candida* homologous with regions of the fungal group within the fungal kingdom with the most divergent DNA sequences and regions of *Candida* incongruous with the human DNA sequence. Primer selection was optimised for melting temperature equivalence, lack of duplex, hairpin, or primer-dimer formation, and internal stability using OLIGO software (Amplify).

### Nested PCR

A number of measures were used to avoid PCR contamination including the use of separate rooms and glassware supplies for PCR set-up and products, aliquoted reagents, positive-displacement pipettes, aerosol-resistant tips and multiple negative controls. DNA samples obtained from frozen CNS tissue were analysed by nested PCR using several primer pairs. To amplify the ITS-1 region, the first PCR was carried out with 4 μl of DNA incubated at 95 °C for 10 min followed by 30 cycles of 45 s at 94 °C, 1 min at 57.3 °C and 45 s at 72 °C. Oligonucleotides used in the first PCR were forward ITS-1 (external 5′): ^1448^5′GTTCTGGGCCGCACGGG 3′^1465^ and reverse ITS-1 (external 3′): ^106R^5′GGCAAAGATTCGATGATT3′^88R^. The second PCR was carried out using one of three primer sets. The second PCR was performed using 0.5 μl of the product obtained in the first PCR and ITS-1 (internal) primers for 30 cycles of 45 s at 94 °C, 1 min at 52 °C and 45 s at 72 °C. Oligonucleotides used were forward ITS-1 (internal 1-5′): ^1781^5′CCTGCGGAAGGATCA3′^1798^ and reverse ITS-1 (internal 1-3′): ^20R^5′GATCCGTTGTTGAAA3′^5R^. As an alternate second PCR, ITS-1 (internal 2) primers were used. The second PCR was carried out with 0.5 μl of the product obtained in the first PCR and ITS-1 (internal 2) primers for 35 cycles of 45 s at 94 °C, 1 min at 55 °C and 45 s at 72 °C. Oligonucleotides used were forward ITS-1 (internal 2-5′): ^1771^5´TCCGTAGGTGAACCTGCGG3′^1790^ and reverse ITS-1 (internal 2-3′): ^50R^5′GCTGCGTTCATCGATGC3′^30R^. The third PCR was carried out using the same conditions as the ITS-1 (internal 1) reaction with the primers forward ITS-1 (internal 3-5′): ^1614^5′CTACTACCGATTGAATGGC3′^1634^ and reverse ITS-1 (internal 3-3′): ^214^5′CAATTCATATTACGTATCGCA3′^193^. Finally, the fourth PCR assay was designed to amplify the ITS-2 region. The first PCR assay was carried out with 4 μl of DNA incubated at 95 °C for 10 min followed by 20 cycles of 45 s at 94 °C, 1 min at 52 °C and 45 s at 72 °C. Oligonucleotides used in the first PCR were forward ITS-2 (external 5′): ^152^5′TTTCAACAACGGATCTC3′^169^ and reverse ITS-2 (external 3′): ^**858**^5′AGTACGGGATTCTCACCCTC3′^**838**^. The second PCR was carried out with 0.5 μl of the product obtained in the first PCR and ITS-2 (internal 1) primers for 25 cycles of 45 s at 94 °C, 1 min at 55 °C, and 45 s at 72 °C. Oligonucleotides used were forward ITS-2 (internal 1-5′): ^274^5′GCATCGATGAAGAACGCAGC3′^295^ and reverse ITS-2 (internal 1-3′): ^572R^5´TCCTCCGCTTATTGATATGC3′^552R^.

The human β-globin gene served as a control for DNA extraction. PCR was carried out with 4 μl of DNA incubated at 95 °C for 10 min and amplified with 42 cycles of 45 s at 94 °C, 1 min at 60 °C and 45 s at 72 °C. Oligonucleotides used were 5′GGTTGGCCAATCTACTCCCAGG 3′ and 3′ GCTCACTCAGTGTGGCAAAG 5′. Amplified DNA products were analysed by agarose gel electrophoresis and stained with ethidium bromide.

## Additional Information

**How to cite this article**: Pisa, D. *et al.* Different Brain Regions are Infected with Fungi in Alzheimer's Disease. *Sci. Rep.* **5**, 15015; doi: 10.1038/srep15015 (2015).

## Supplementary Material

Supplementary Information

## Figures and Tables

**Table 1:** Fungal species present in frozen CNS samples from AD patients detected by PCR.

Species	EFC	CEH	ERH	CP
*Candida albicans*				2
*Candida ortholopsis*		2		
*Candida tropicalis*			2	
*Cladosporium*			2	2
*Malassezia globosa*	1	1		1
*Malassezia restricta*				4
*Neosartorya hiratsukae*		4		
*Phoma*	4			
*Sacharomyces cerevisae*		2; 3		
*Sclerotinia borealis*			3	1;3

EFC: External frontal cortex; CEH: Cerebellar hemisphere; ERH: Entorhinal cortexI/hippocampus; CP: Choroid plexus. The numbers in the Table indicate the PCR number which were positive for a given species.

PCR 1: ITS-1 (external) + ITS-1 (internal 1).

PCR 2: ITS-1 (external) + ITS-1 (internal 2).

PCR 3: ITS-1 (external) + ITS-1 (internal 3).

PCR 4: ITS-2 (external) + ITS-2 (internal 1).
