# Supplementary material for: Different Brain Regions are Infected with Fungi in Alzheimer’s Disease
Source: Sci Rep. 2015 Oct 15;5:15015. doi: 10.1038/srep15015 (PMC4606562; doi:10.1038/srep15015)
Supplement: Supplementary Information [file srep15015-s1.pdf]

DIFFERENT BRAIN REGIONS ARE INFECTED WITH FUNGI IN ALZHEIMER'S DISEASE

Diana Pisa[1], Ruth Alonso[1], Alberto Rábano[2], Izaskun Rodal[2] and Luis Carrasco[1*]

[1]Centro de Biología Molecular "Severo Ochoa". c/Nicolás Cabrera, 1. Universidad Autónoma de Madrid. Cantoblanco. 28049 Madrid. Spain.

[2]Department of Neuropathology and Tissue Bank, Unidad de Investigación Proyecto Alzheimer, Fundación CIEN, Instituto de Salud Carlos III, Madrid. Spain.

[*]Corresponding author

Email address: lcarrasco@cbm.csic.es , telephone number: +34 91 497 84 50

Running title: Fungal infection in Alzheimer's disease

Key words: Neurodegenerative disease; Fungal infection; Endomycosomes; Fungal DNA; Central nervous system

Supplementary Figure 1. Immunohistochemistry analysis of different fields of EFC from patient AD1 using anti-*C. glabrata* antibodies. Three different fields of this section are shown. Different magnifications of the same field are shown from left to right. DAPI is shown in blue. Anti-*C. glabrata* appears in green and Tau_{T100} in red.

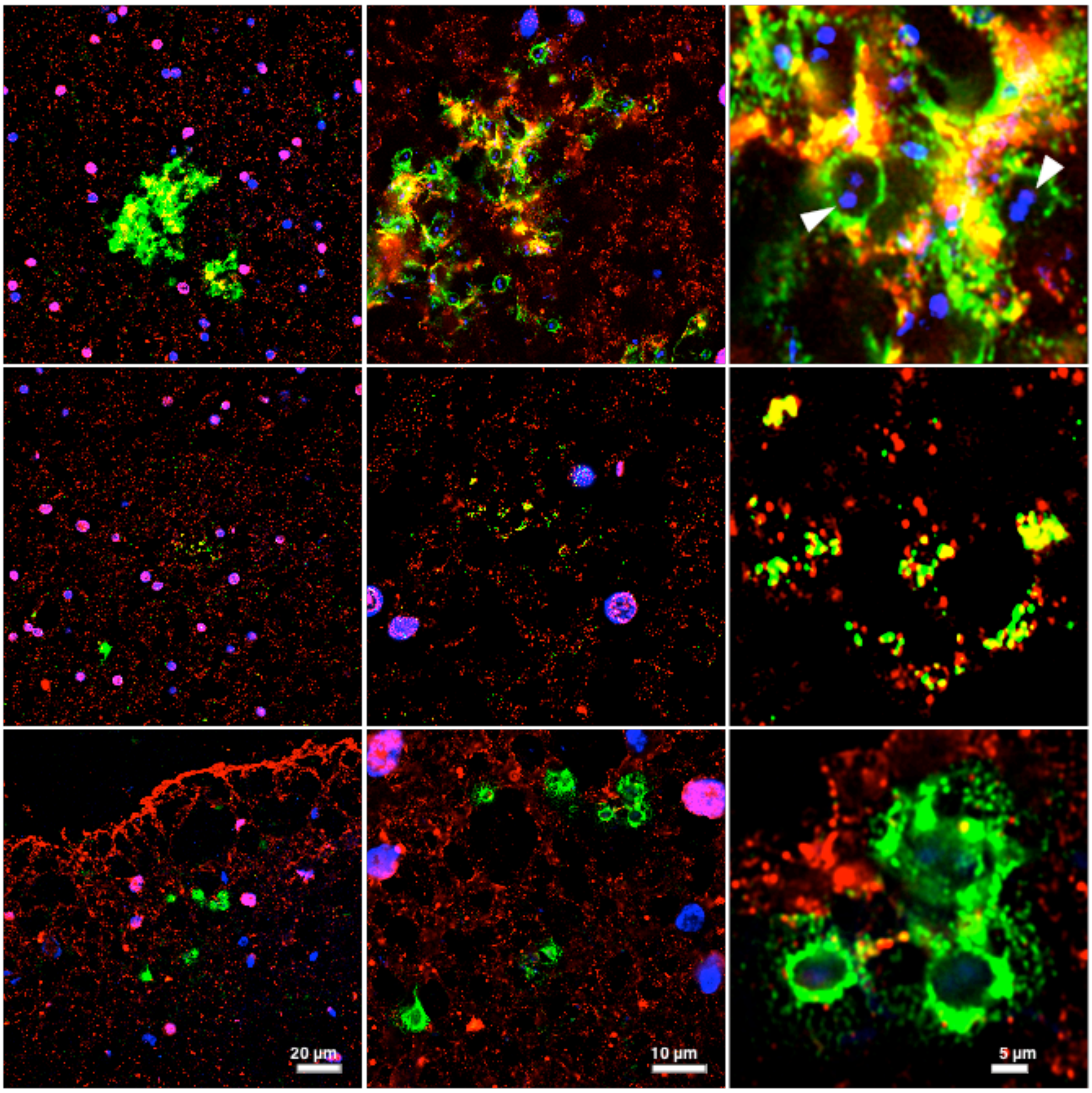

Supplementary Figure 2. Immunohistochemistry analysis of different fields of ERH from patient AD1 using anti-*C. glabrata* antibodies. Three different fields of this section are shown. Different magnifications of the same field are shown from left to right. DAPI is shown in blue. Anti-*C. glabrata* appears in green and Tau_{T100} in red.

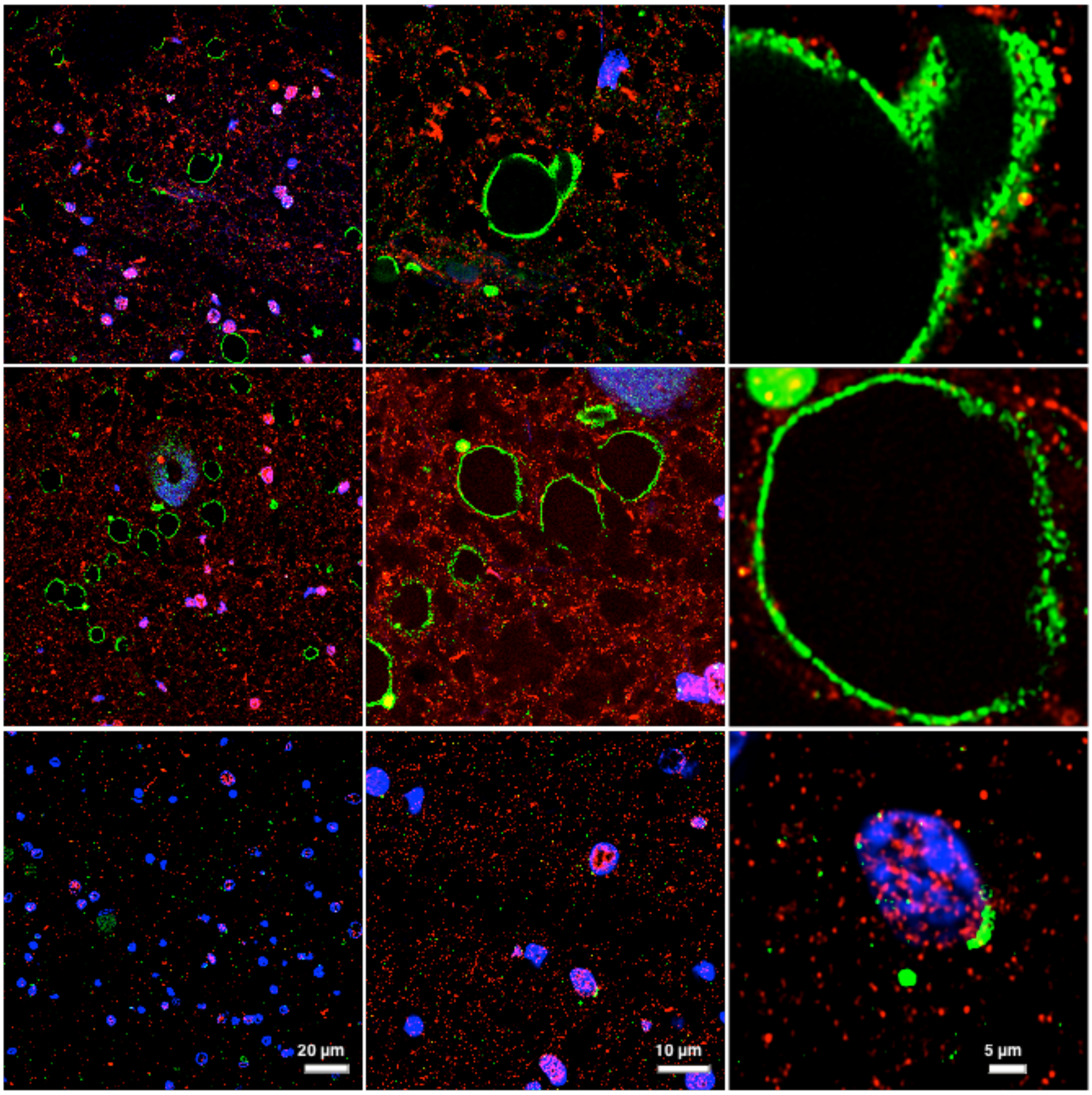

Supplementary Figure 3. Immunohistochemistry analysis of different fields of CEH from patient AD1 using anti-*C. glabrata* antibodies. Three different fields of this section are shown. Different magnifications of the same field are shown from left to right. DAPI is shown in blue. Anti-*C. glabrata* appears in green and Tau_{T100} in red.

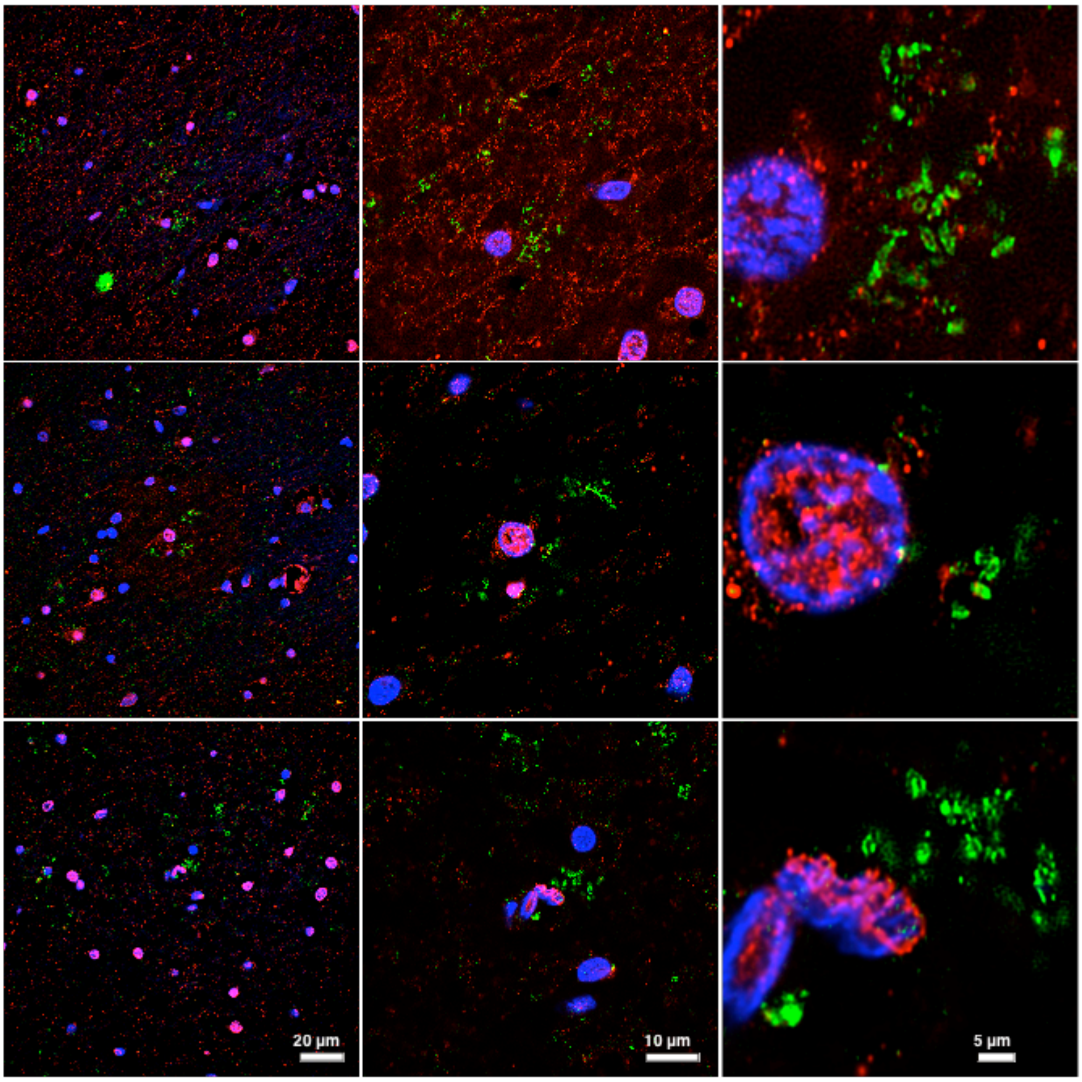

Supplementary Figure 4. Western blot analysis of proteins extracted from CNS regions of patient AD1 and C1. Tissue samples from EFC, CEH and ERH from patient AD1 and C1 were homogenized and proteins were obtained as described in Materials and Methods. The three CNS samples from patients AD1 and C1 were separated by SDS-PAGE and transferred to a nitrocellulose membrane, which was incubated with anti-*C. albicans* antibodies (1:1000 dilution) (left panel). As a control, *C. albicans* proteins were also tested alone (middle lane) and after addition of this yeast proteins to CNS samples from patient AD1 and C1 (right panel). MW (kd) markers are indicated on the left.

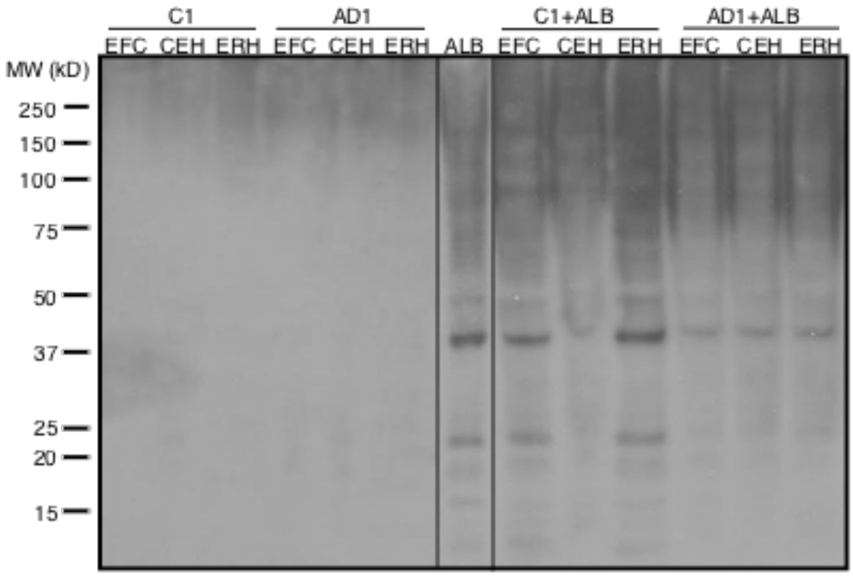

Supplementary Figure 5. Immunohistochemistry analysis of ERH from different control individuals using anti-*C. glabrata* antibodies. ERH sections from ten different control individuals were processed for double immunofluorescence analysis. Samples were incubated with two primary antibodies: anti-*C. glabrata* (green) and anti-α-tubulin (red). Immunohistochemistry was carried out as described in Materials and Methods. DAPI appears in blue. Scale bar: 20 µm.

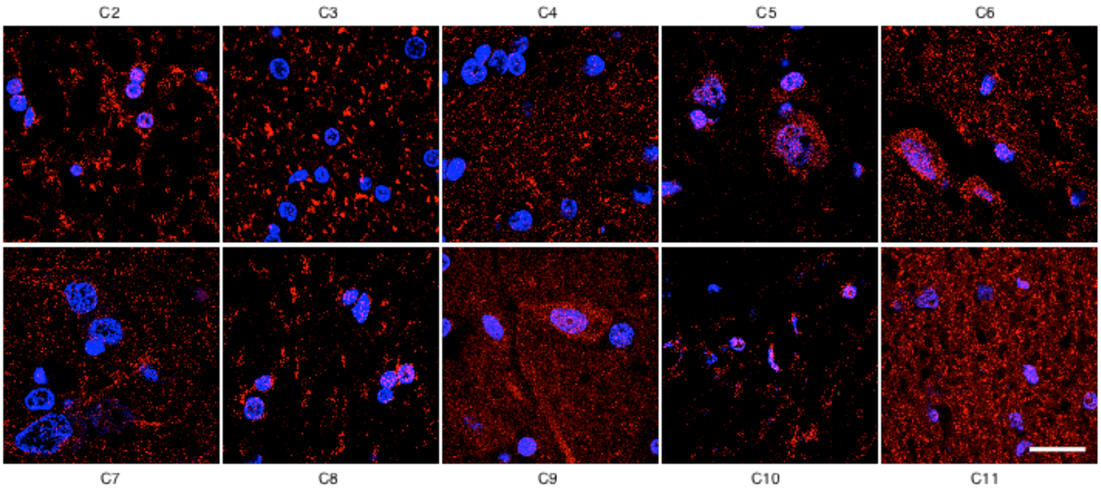

Supplementary Figure 6. Fungal bodies in CP of different AD patients. CP of patient C1 with anti-*C. famata,* anti-*C. albicans,* anti-*P. betae* antibodies as indicated on

the panels and anti-human neurofilament in all panels. CP of AD 12, AD 13 and AD 14. Panels A, D, G: AD 12. Panels B, E, H: AD 13. Panels: C, F, I: AD 14. Panels A, B, C: primary antibodies employed were anti-*C. glabrata* and anti-Tau_{T100}. Panels D, F: primary antibodies employed were anti-*C. famata* and anti-human neurofilament. Panel E: primary antibodies employed were anti-*C. albicans* and anti-human neurofilament. Panels G, H, I: primary antibodies employed were: anti-*P. betae* and anti-human neurofilament. Anti-fungus antibodies are shown in green. Anti-human neurofilament and anti- Tau_{T100} are shown in red. DAPI appears in blue. Scale bar: 10 μm.

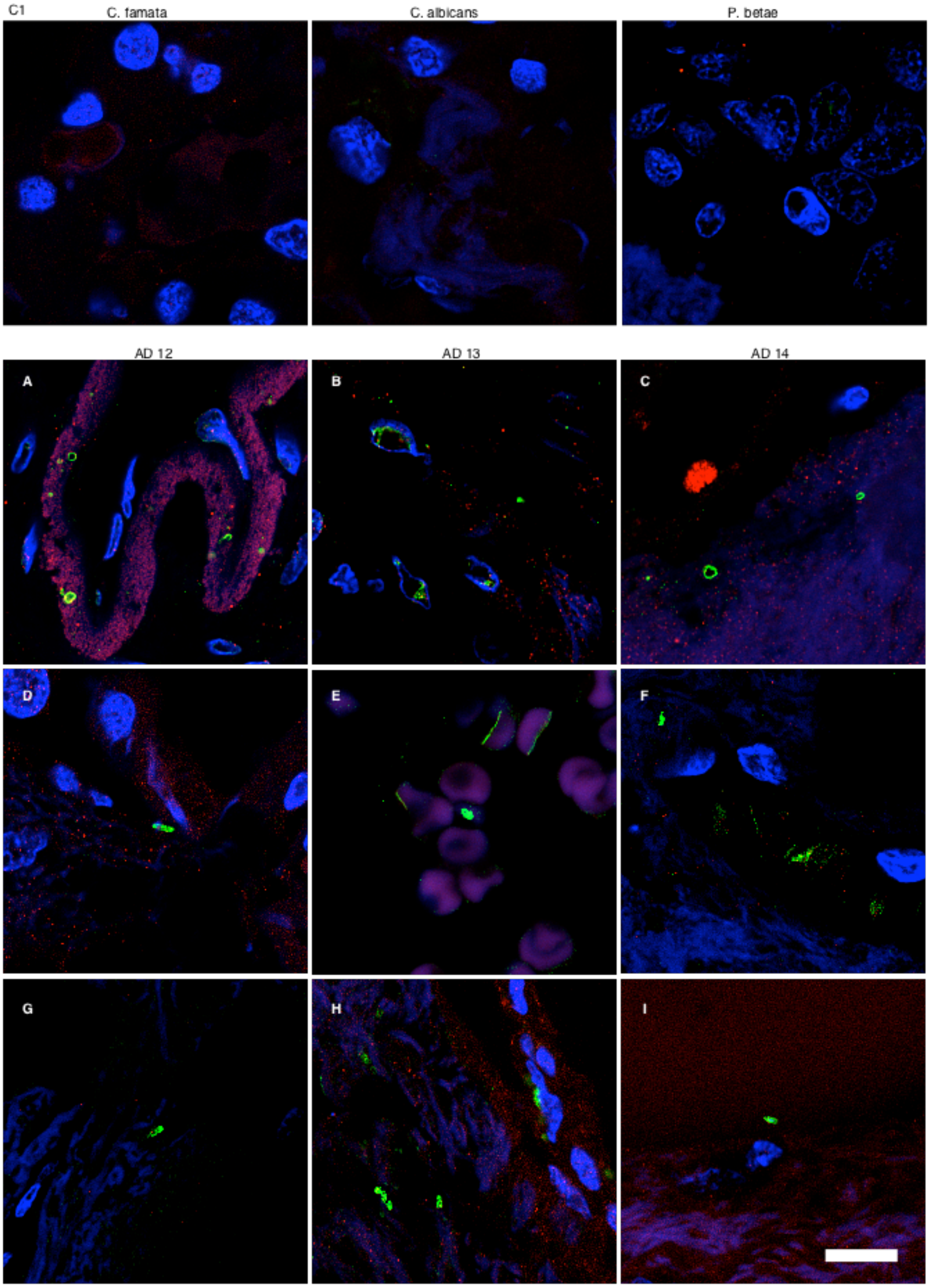

Supplementary Figure 7. PCR analysis of different brain regions from patient AD1 and C1. A) Schematic representation of fungal rRNA genes (18S, 5.8S and 28S), and ITS-1 and ITS-2 sequence. Location of the primers employed for PCR amplification: External ITS-1 primers employed in the first PCR to amplify ITS-1 and internal primers

(1,2,3) employed in the second PCR. The ITS-2 region was first amplified with external ITS-2 primers employed in the first PCR followed by a second PCR using primers internal 1´ employed in the second PCR. B) PCR was carried out as described in Materials and Methods using DNA of brain samples obtained from AD1 and C1. The primers employed were external ITS-1 for the first round PCR and primers internal 1 for the second round. C) PCR analysis of DNA extracted from the samples tested in panel B using human β-globin oligonucleotide primers. After PCR, the samples were separated on agarose gels and stained with ethidium bromide. DNA size markers are shown on the right. Control +: DNA from Hela cells; Control PCR: PCR without DNA; CE: Control of DNA extraction without brain DNA. EFC: External frontal cortex; CEH: Cerebellar hemisphere; ERH: Entorhinal cortex/hippocampus; CP: Choroid plexus.

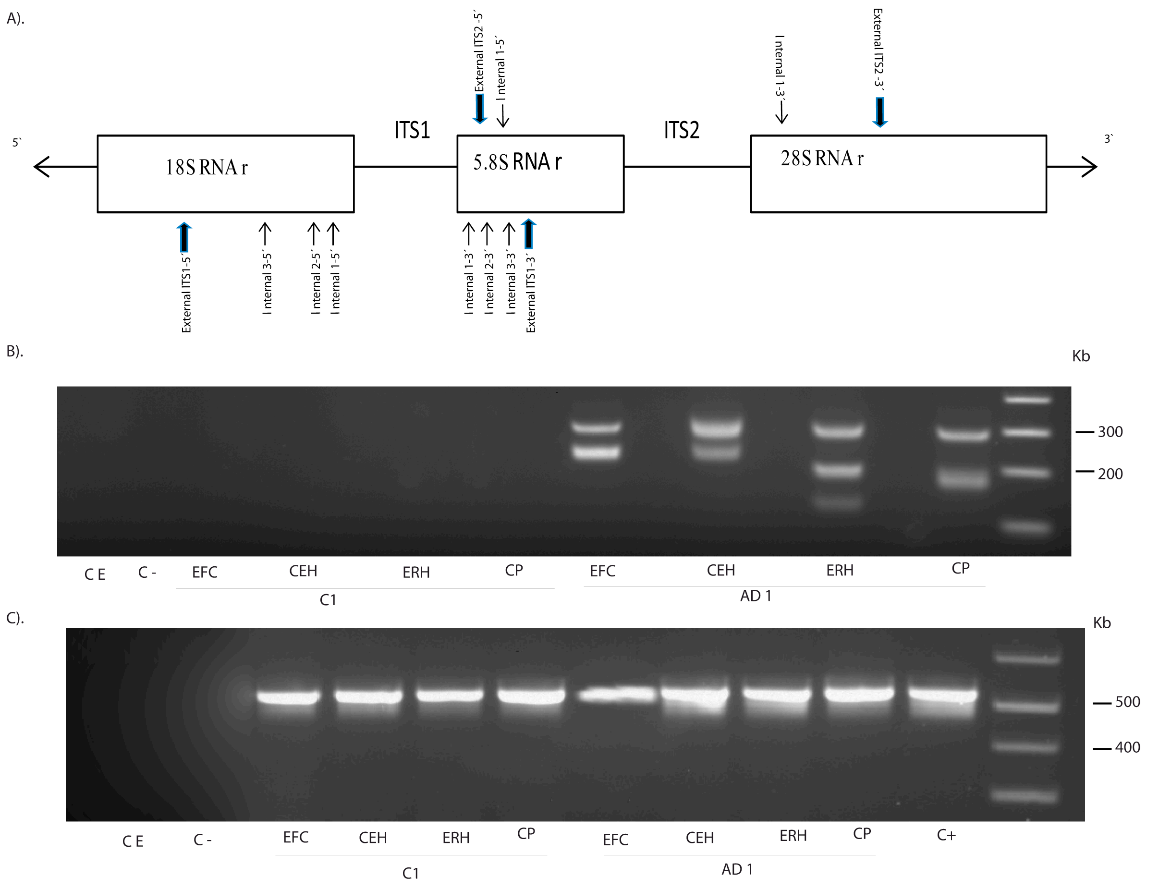

Supplementary table 1. Age and gender of AD patients and control individuals

analyzed in this work.

EFC: external frontal cortex; CEH: cerebellar hippocampus; ERH: entorhinal cortex/hippocampus; CP: choroid plexus. M: male. F: female.

**PATIENT**	**ZONE**	**AGE**	**GENDER**
AD1	EFC,CEH,ERH,CP	83	M
AD2	ERH	80	M
AD3	ERH	84	M
AD4	ERH	79	M
AD5	ERH	81	M
AD6	ERH	87	F
AD7	ERH	92	F
AD8	ERH	81	F
AD9	ERH	87	M
AD10	ERH	86	M
AD11	ERH	62	F
AD12	CP	87	F
AD13	CP	77	F
AD14	CP	87	F
**CONTROL**	**ZONE**	**AGE**	**GENDER**
C1	EFC,CEH,ERH,CP	77	M
C2	ERH	56	F
C3	ERH	48	M
C4	ERH	63	F
C5	ERH	78	F
C6	ERH	55	M
C7	ERH	62	M
C8	ERH	84	F
C9	ERH	37	M
C10	ERH	54	M
C11	ERH	59	F
